# The Effects of Self-Regulated Mathematics Instruction with Integrated Cognitive and Metacognitive Strategies on High School Students’ Self-Regulation Skills, Attitudes Toward Mathematics, and Motivation in Mathematics

**DOI:** 10.3390/bs16101843

**Published:** 2026-10-08

**Authors:** Rukiye Aydoğan

**Affiliations:** Faculty of Sport Sciences, Aydın Adnan Menderes University, 09010 Aydın, Türkiye; rukiye.aydogan@adu.edu.tr

**Keywords:** self-regulated learning, cognitive strategies, metacognitive strategies, mathematics education, mathematics motivation, attitudes toward mathematics, high school students

## Abstract

(1) Background: In this study, the effect of the Structured Mathematics Teaching Plan Integrating Cognitive and Metacognitive Strategies Based on Self-Regulated Learning (MLP-SRL) on 11th-grade students’ self-regulation skills, attitudes toward mathematics, and motivation in mathematics was examined. (2) Methods: A mixed-methods approach was used. In the quantitative component, a quasi-experimental non-equivalent control group design was used, while semi-structured interviews were employed in the qualitative component. During the eight-week (48 class hours) intervention, the experimental group received instruction integrating cognitive and metacognitive strategies based on Zimmerman’s cyclical model, while the control group followed the current curriculum. Data were collected using the Self-Regulation Scale, the Attitude Toward Mathematics Scale, and the Mathematics Motivation Scale; quantitative data were analyzed using mixed ANOVA, while qualitative data were analyzed using content analysis. (3) Results: The MLP-SRL intervention significantly improved students’ self-regulation skills, attitudes toward mathematics, and motivation. Qualitative results indicated improvements in metacognitive monitoring, strategic problem-solving, goal setting, self-reflection, and conceptual error analysis. Students also reported reduced mathematics anxiety and the use of cognitive and metacognitive strategies across learning tasks. (4) Conclusions: Integrating cognitive and metacognitive strategies into self-regulated mathematics instruction supports students’ cognitive and affective development while strengthening strategy use, self-regulation, attitudes toward mathematics, and motivation.

## 1. Introduction

Mathematics is recognized as one of the foundational disciplines for developing higher-order cognitive skills such as problem-solving, analytical thinking, and logical reasoning. However, mathematics education remains one of the areas where students face the greatest challenges (Li & Schoenfeld, 2019; Nelson & Powell, 2018). Research shows that difficulties encountered in mathematics learning cannot be explained solely by cognitive abilities; affective characteristics such as students’ motivation toward mathematics, attitudes, and anxiety levels also play a significant role in learning (Gabriel et al., 2020; Zamir et al., 2022/2023). In particular, low motivation, negative attitudes toward mathematics, and high levels of math anxiety can undermine students’ engagement with mathematics tasks, the effort they exert in the face of difficulties, and the continuity of their learning processes (Fiorella et al., 2021; Gabriel et al., 2020). For this reason, focusing solely on cognitive gains in mathematics instruction is no longer sufficient; supporting the motivational and self-regulatory mechanisms that guide students’ learning is increasingly important.

In the fields of psychology and educational sciences, self-regulation is defined as the process by which an individual plans, monitors, and controls their emotions, thoughts, and behaviors in a deliberate, conscious manner to achieve their goals (Pintrich, 2000; Zimmerman, 2000). The concept of self-regulated learning (SRL) emerges from adapting this general framework to learning processes. Self-regulated learning is defined as a cyclical learning process in which students set their own learning goals, systematically employ cognitive, metacognitive, and motivational strategies to achieve these goals, monitor their learning processes, and make necessary adjustments by evaluating the results they obtain (Cleary & Kitsantas, 2017). This approach shifts students from passive recipients of learning to active learners who manage their own learning. In mathematics education, this process supports students—particularly during problem-solving activities—in selecting appropriate cognitive strategies (such as formula deconstruction and representational modeling), monitoring the solution process through metacognitive evaluation, persevering in the face of difficulties, analyzing errors, and developing alternative solution paths when necessary (Tee et al., 2020). Therefore, self-regulated learning is not merely an approach that supports students’ learning processes; it is also a fundamental learning skill that enables students to take ownership of their learning (Říčan et al., 2022).

Students’ ability to develop sustainable motivation across various academic subjects and adopt positive attitudes toward their learning depends largely on teaching and the effective use of self-regulation skills, which enable them to manage their learning processes. The literature indicates that students who learn self-regulation strategies (such as goal setting, planning, and self-monitoring) through structured processes develop greater control over their learning processes, which in turn contributes to a significant increase in their self-efficacy, task value, and levels of intrinsic motivation (Léon et al., 2015; Fiorella et al., 2021). Conversely, it has been shown that instructional processes in which self-regulation mechanisms are not sufficiently supported can increase students’ test anxiety. Due to limited self-assessment and reflective thinking processes, they can lead students to give up more easily during problem-solving and complex tasks; by negatively affecting affective characteristics, they can hinder students’ engagement and learning processes (Gabriel et al., 2020; Zamir et al., 2022/2023). Conversely, the systematic integration of self-regulated learning processes into instructional settings fosters students’ strategic competencies across various disciplines; this sense of competence, in turn, contributes to a reduction in negative perceptions of academic fields and the development of positive emotions and attitudes (Cleary & Kitsantas, 2017; Hıdıroğlu & Hıdıroğlu, 2026). Therefore, self-regulation training should not be viewed as a secondary element from which affective outcomes are expected to emerge spontaneously, but rather as a crucial learning process that supports students’ cognitive functioning, motivational beliefs, and academic attitudes across various courses.

Although there has been an increase in intervention studies on self-regulated learning in recent years, a significant portion of these studies have been conducted with college students and have primarily focused on academic achievement (Cohaila, 2022; Zheng & Sun, 2024; Jansen et al., 2019; Theobald, 2021). It is evident that experimental studies at the high school level are limited; in particular, relatively few studies have examined self-regulation, mathematics motivation, and attitudes toward mathematics together within the same instructional intervention (Tee et al., 2018, 2020; Cohaila, 2022; Hıdıroğlu & Hıdıroğlu, 2026). Moreover, systematic reviews of self-regulated learning interventions in mathematics have emphasized the importance of cognitive, metacognitive, and motivational strategies, while also indicating the need for further research on how these components can be effectively integrated within mathematics instruction (Wang & Sperling, 2020).

Accordingly, the effect of instruction delivered using the Mathematics Lesson Plan Based on Self-Regulated Learning with Integrated Cognitive and Metacognitive Strategies (MLP-SRL)—a structured mathematics teaching plan grounded in self-regulated learning—on 11th-grade students’ self-regulation, attitudes towards mathematics, and motivation for mathematics was examined using a mixed-methods approach. The hypotheses and the qualitative research question formulated within the scope of the study are as follows:

**H1.**  *Instruction using MLP-SRL significantly increases the self-regulation scores of students in the experimental group compared to the control group.*

**H2.**  *Instruction using MLP-SRL significantly increases the math attitude scores of students in the experimental group compared to the control group.*

**H3.**  *Training conducted using MLP-SRL significantly increases the mathematics motivation scores of students in the experimental group compared to the control group.*

Q_1_: “What are the students’ evaluations of the experimental intervention process and their own development during this process?”

## 2. Materials and Methods

### 2.1. Model

In this study, a mixed-methods approach was adopted, and an intervention design was used to comprehensively examine the effects of the Mathematics Lesson Plan Based on Self-Regulated Learning with Integrated Cognitive and Metacognitive Strategies (MLP-SRL) program on students (Creswell & Plano Clark, 2018). In the quantitative phase, a quasi-experimental, nonequivalent control group design with two intact classrooms was implemented; students’ self-regulation skills, attitudes toward mathematics, and motivation levels were assessed. To interpret the results more comprehensively, semi-structured interviews were conducted in the second phase, and the qualitative data were analyzed using content analysis (Bryman, 2016). Finally, at the interpretation stage, quantitative and qualitative results were integrated to evaluate the effectiveness of the MLP-SRL intervention alongside the participants’ experiences (Fetters, 2013; Tashakkori & Teddlie, 2021). The research design is presented in Table 1.

Table 1 summarizes the stages of the research process. The process began with the administration of the Self-Regulated Learning Scale, the Attitude Toward Mathematics Scale, and the Mathematics Motivation Scale as pre-tests to both the experimental and control groups. Subsequently, over eight weeks, MLP-SRL-based instructional activities were implemented in the experimental group, while the control group followed the existing curriculum. At the end of the process, the same measurement instruments were administered again as post-tests, followed by semi-structured interviews with students in the experimental group, and changes in scores, alongside qualitative findings, were evaluated using appropriate statistical and qualitative analyses (Büyüköztürk et al., 2014). Data triangulation was achieved by collecting quantitative and qualitative data. At various stages of the study, input was sought from three experts in educational sciences and mathematics education regarding the measurement tools and the intervention plan. The aim was to strengthen the generalizability of the findings by providing a detailed explanation of the research design, the implementation process, participant characteristics, and data collection procedures.

### 2.2. Study Group

The sample for the quantitative study consisted of 38 11th-grade students at a public high school in Turkey with a medium socioeconomic status. The participants were enrolled in two existing classes. The two existing classes were randomly assigned as the experimental and control groups, with the classroom serving as the unit of allocation. The experimental group consisted of 20 students (11 girls, 9 boys), while the control group consisted of 18 students (10 girls, 8 boys). There are no students with special education needs in either the experimental or control groups. The school where the study was conducted was selected using the criterion sampling method.

Interviews were conducted with students participating in the experimental phase to collect qualitative data. These participants were selected using the maximum diversity sampling method, taking into account differences in gender and mathematics achievement levels. During the selection process, a list of participants was first compiled based on students’ regular participation in the experimental program and their first-semester mathematics scores, while also accounting for different mathematics achievement levels and genders. Students were invited to the interviews in the order of this list, and interviews were conducted with those who voluntarily agreed to participate. In total, interviews were conducted with 7 students (4 female and 3 males; 2, 3, and 2 students from the low, medium, and high mathematics achievement levels, respectively). All qualitative participants demonstrated regular participation in the experimental application process. This selection aimed to ensure that the diverse student profiles in the experimental group were reflected in the qualitative dataset.

### 2.3. Measure

#### 2.3.1. Self-Regulation Scale for Adolescents (SRSA)

The Adolescent Self-Regulation Scale, developed by Kaşıkçı and Öğülmüş (2023), assesses adolescents’ self-regulation skills. AFA and DFA results indicated that the scale has a unidimensional structure consisting of 11 items, explaining 51% of the total variance. The scale, which was found to have adequate item discrimination, also demonstrated measurement invariance across genders. The scale’s Cronbach’s Alpha, McDonald’s Omega, and CR values are 0.90, 0.89, and 0.89, respectively. The findings indicate that the scale possesses adequate psychometric properties for measuring high school students’ self-regulation skills.

#### 2.3.2. Mathematics Course Attitude Scale (MCAS)

The scale developed by Eskici et al. (2017) consists of 13 items and has a unidimensional structure. In the AFA results, the KMO value was 0.94, and the Bartlett test was significant $\left(\chi^{2}=2400.03,\;p<0.001\right)$. Item-factor loadings were found to range from 0.47 to 0.88. DFA, on the other hand, showed that the unidimensional structure had acceptable fit indices ($\chi^{2}=$ 3.178, CFI = 0.913, TLI = 0.927, RMSEA = 0.0092). The fact that the scale’s Cronbach’s Alpha and composite reliability (CR) coefficients are above 0.90 demonstrates that it is a psychometrically valid and reliable measurement tool.

#### 2.3.3. Mathematics Motivation Scale (MMS)

The Mathematics Motivation Scale, developed by Kesici (2018), is a 12-item, 5-point Likert-type instrument consisting of three subscales: Goal Setting, Expectation-Value, and Self-Efficacy. The AFA results showed that the scale explained approximately 65% of the total variance and that the Cronbach’s Alpha coefficient was 0.87. DFA findings revealed that the model possesses acceptable fit indices ($\chi^{2}$/*df* = 2.11, RMSEA = 0.075, GFI = 0.92, AGFI = 0.87, NFI = 0.90, CFI = 0.94). In conclusion, the scale is valid and reliable for measuring high school students’ motivation in mathematics.

#### 2.3.4. Semi-Structured Interview Form

Following the experimental intervention, a semi-structured interview form was developed to examine the experimental group students’ use of cognitive and metacognitive strategies, self-regulation processes, mathematics experiences, and views on the MLP-SRL application. Prepared with input from three experts (in educational sciences and mathematics education) to ensure content and face validity, the form consists of nine core questions and follow-up probing questions. Face-to-face individual interviews with the seven purposively selected students in the experimental group were audio-recorded, with the consent of the participants and their parents, and lasted an average of 15 min each. The interviews were conducted in a quiet and appropriate setting so that students could feel comfortable and safe. To prevent potential grade-related anxiety from influencing students’ responses, the interviews were conducted after the grading process was complete. In addition, students were given the opportunity to express their views without fear of evaluation, in ways that reflected their own experiences. To ensure the collection of objective data and to minimize potential social desirability bias, all semi-structured interviews were conducted by the researcher.

### 2.4. Experimental Procedure and Intervention

The study was conducted during the second semester of the 2025–2026 academic year as part of the 11th-grade mathematics course. Prior to the experimental implementation, the researcher provided training to the mathematics teacher who would conduct the experiment on the “Mathematics Lesson Plan Based on Self-Regulated Learning Integrated with Cognitive and Metacognitive Strategies” (MLP-SRL).

To ensure that the intervention could be carried out as planned by the implementer, the mathematics teacher received preparatory training on the MLP-SRL program from the researcher prior to implementation. Because the researcher is an expert in educational sciences and the intervention was conducted in a high school mathematics class, the intervention was implemented by a high school mathematics teacher. Accordingly, measures were taken to ensure that the implementing teacher was prepared to conduct the lessons in a manner consistent with the context of mathematics instruction and the MLP-SRL program. The training was conducted in person at the school where the intervention was to take place and with the teacher. The training first covered the theoretical foundations of MLP-SRL and Zimmerman’s cyclical self-regulated learning model; it then explained how the three core stages outlined in the lesson plan—pre-reflection, performance/volitional control, and self-reflection—would be implemented in the classroom. The guide explains how teachers should guide students, particularly in setting learning goals and engaging in strategic planning, using cognitive and metacognitive strategies, monitoring their learning processes, and evaluating their own performance at the end of the lesson. In addition, explanations are provided for the implementation of the problem-solving activities, including analysis of formulas and procedural steps, use of graphical/geometric representations, mathematical error analysis, problem-solving with peers, and end-of-lesson assessment activities included in the lesson plan. Thus, the aim was for the teacher to become proficient not only in the lesson plan’s content but also in implementing the self-regulated learning approach on which the plan is based. During the training, the teacher’s questions about the program were addressed, and implementation uncertainties were resolved through mutual discussion grounded in application examples and potential classroom scenarios. Following the initial preparatory training, the first lesson was conducted. Afterward, the implementation process was reviewed together with the teacher, and the program’s phases, the teacher’s role in implementation, and key points regarding classroom practices were reassessed.

To ensure fidelity to the intervention, a checklist of key implementation steps from the MLP-SRL lesson plan was used. The checklist is structured under five main steps to outline the key implementation steps for each lesson. These steps include (1) implementing the pre-reflection phase, (2) setting learning objectives and conducting strategic planning, (3) using cognitive and metacognitive strategies, (4) monitoring the performance process, and (5) conducting self-reflection and end-of-lesson evaluation activities. At the end of each lesson, the teacher evaluated the activities carried out using a checklist and indicated whether the relevant steps had been implemented. Additionally, weekly meetings (sometimes in person, sometimes by phone) were held with the teacher throughout the intervention process; during these meetings, the activities for the relevant week were reviewed in accordance with the lesson plan and checklist, any difficulties encountered were addressed, and necessary explanations regarding the activities for the following week were provided.

During the intervention, care was taken to ensure that the practical steps outlined in the lesson plan were completed within the scheduled class time. However, there were instances where the two-week class period proved insufficient to complete all planned activities. For example, in some classes where the self-reflection and end-of-class evaluation phases could not be completed within the class period, evaluation activities that students could complete individually were assigned as homework and followed up on in the next class. In this way, rather than completely omitting the relevant phase of the program due to time constraints, the planned learning process was preserved. Such situations were evaluated during weekly teacher meetings, and necessary adjustments were made to ensure more effective use of class time in subsequent sessions.

To control for potential external variables stemming from the teacher, lessons in both the experimental and control groups were taught by the same teacher. While classroom instruction and the MLP-SRL intervention were conducted by the trained teacher, the subsequent data collection process—including qualitative interviews—was conducted independently by the researcher. Similarly, to ensure the collection of standardized, objective data, the quantitative pre-test and post-test assessment tools (self-regulation, attitude, and motivation scales) were administered directly by the researcher to both groups. The experimental process lasted eight weeks, totaling 48 class hours; while lessons in the experimental group were conducted in accordance with the MLP-SRL, lessons in the control group followed the existing curriculum. Prior to the study, written informed consent forms were distributed to all participants and signed by their legal guardians.

#### 2.4.1. Mathematics Lesson Plan Based on Self-Regulated Learning with Integrated Cognitive and Metacognitive Strategies (MLP-SRL)

High school mathematics instruction requires abstract thinking, problem-solving, and higher-order cognitive processes. Students’ success in mathematics depends not only on their knowledge and skills but also on their attitudes toward mathematics, motivation, and ability to regulate their learning processes. Because mathematics tasks require students to analyze tasks, select appropriate strategies, monitor their understanding, and evaluate their solutions, self-regulated learning is particularly relevant to mathematics instruction. Within this framework, cognitive and metacognitive strategies enable students to actively regulate their learning by supporting strategic action, monitoring, and evaluation throughout the learning process. Accordingly, the present study implemented a structured mathematics teaching plan designed to support students’ goal setting, strategic cognitive and metacognitive processes, and self-reflection as they engage with complex mathematic problems.

Self-regulated learning is an approach that describes how students take an active role in their learning processes and is addressed through various theoretical models (Boekaerts, 1999; Pintrich, 2000; Zimmerman, 2000). These models collectively emphasize the processes of goal setting, the use of appropriate cognitive and behavioral strategies, and the evaluation of learning performance. MLP-SRL used in this study was adapted to the learning outcomes of an 11th-grade mathematics course, based on Zimmerman’s (2000) cyclical self-regulated learning model and utilizing Aydoğan’s (2018) self-regulated learning-based lesson flowchart. The model consists of the stages of anticipation, performance/volition control, and self-reflection; it encompasses the dimensions of motivation, method, time management, behavior control, physical environment, and social environment (Zimmerman, 2000). In line with this structure, students’ abilities to set goals, select cognitive and metacognitive strategies, monitor the process, and evaluate results in mathematics tasks were supported.

During implementation, students’ self-regulation skills were developed in accordance with the three stages of the MLP-SRL. In the anticipation phase, students set goals, assessed their self-efficacy, and planned learning strategies; in the performance/willpower control phase, they used appropriate strategies, managed their attention and time, sought support from peers or teachers when necessary, and adjusted their strategies accordingly; and in the self-reflection phase, they evaluated their performance, the strategies used, and their goals. The eight-week intervention was integrated into the 11th-grade mathematics curriculum learning outcomes; the intervention’s reliability was supported by process monitoring and lesson plan reviews. The detailed content and implementation steps of the MLP-SRL are presented in Appendix A.

The course consists of two units and four subtopics. In the first unit, “Solid Figures (Spatial Geometry),” the following topics were covered in order: right circular cylinders, right circular cones, spheres, and problem-solving and application exercises involving solid figures. The second unit, Probability, covers conditional probability, dependent and independent events, compound events, and experimental and theoretical probability, in that order. Weekly math classes were conducted in two consecutive periods (80 min total). In the first phase of the MLP-SRL, guidance, explanations, and cognitive and metacognitive questions were included to support students in preparing for the lesson and establishing connections between their prior learning and the new topic. This section lasted an average of 15 min. The second phase consisted of the core application phase, in which the strategies were directly applied by integrating them with the mathematics content; this phase lasted an average of 45 min. In this section, one or more activities were conducted depending on the week’s mathematics content and learning objective. Details regarding the activities and materials used are provided in Appendix B. The third phase lasted 20 min. To support students in evaluating their learning processes and reinforcing their learning, cognitive and metacognitive questions were primarily used; depending on the lesson content and students’ needs, short assessment activities, mini-tests, self-assessment, and reviews of problem-solving processes were also incorporated.

#### 2.4.2. Researcher Reflexivity and Positionality

In this study, the researcher served a dual methodological role: providing training to the classroom teacher on the MLP-SRL application and independently conducting data collection. The researcher did not teach the students, nor did the teacher play any role in conducting the student interviews or interpreting the data. Aware that their position and relationship with participants could influence responses through power imbalances, social desirability bias, or researcher expectations, the researcher avoided leading statements during data collection. Students were informed that their interview responses would be kept confidential and would not affect their academic evaluations. The semi-structured interviews were conducted independently of the classroom teacher; interview data were anonymized during the analysis and reporting phases, and no information that could reveal the participants’ identities was used.

### 2.5. Data Analysis

#### 2.5.1. Quantitative Data Analysis

IBM SPSS 27 and MS Excel were used to analyze the quantitative data. The gender distribution of the participants was determined using frequency analysis, and a significance level of 0.05 was adopted for all analyses. Normality assumptions were examined to determine the appropriate analytical methods. Since the Shapiro–Wilk test yields more reliable results when the sample size is less than 50 (Büyüköztürk, 2019; Pituch & Stevens, 2016), the Shapiro–Wilk test, along with skewness and kurtosis values, was used to assess normality. The findings are presented in Table 2.

An examination of Table 2 reveals that, according to the results of the Shapiro–Wilk test, scores on the Self-Regulation Scale and the Mathematics Motivation Scale were normally distributed (*p* > 0.05), whereas the test result for scores on the Attitude Toward Mathematics Scale was significant (*p* < 0.05). However, the literature indicates that normality tests in small samples may be influenced by sample size and should be evaluated in conjunction with skewness and kurtosis values (Field, 2018; Ghasemi & Zahediasl, 2012). Furthermore, the applicability of parametric tests is supported when skewness and kurtosis values fall within a range of approximately ±1.5 (George & Mallery, 2016; Tabachnick & Fidell, 2013). Additionally, the analysis of variance (ANOVA) is widely recognized to be robust against moderate departures from normality, particularly when group sizes are nearly equal (Field, 2018; Tabachnick & Fidell, 2013). Accordingly, the data were deemed suitable for parametric analysis; homogeneity of variances was examined using the Levene test, and a 2 × 2 mixed-measures ANOVA was used to determine time × group effects.

#### 2.5.2. Qualitative Data Analysis

In this study, qualitative data were collected through semi-structured interviews and analyzed using content analysis. This method ensures a comprehensive and in-depth examination of the data (Bryman, 2016). Throughout this process, the findings derived inductively from the data were considered alongside the conceptual frameworks that constitute the study’s quantitative dimension.

In this study, the sample size was not predetermined; rather, the research was conducted based on data saturation, in keeping with the nature of qualitative research. As the fieldwork and interviews progressed—particularly by the time of the interview with the sixth participant—it became apparent that the experiences, challenges, and strategies described by the students largely overlapped with those in previous interviews. It was observed that the data collection process had reached saturation at this point; to confirm this, an additional interview was conducted with a seventh participant. In this final interview, no new statements emerged, and the existing narratives were repeated. This indicated that the data collection process had reached a natural saturation point, and the data were sufficient for analysis.

After the interviews were completed, the recordings were transcribed verbatim into written text to preserve the participants’ statements. In the analysis and reporting process, a hybrid approach combining inductive and deductive methods was adopted. During the inductive coding phase, students’ statements were derived as open codes based on the raw data, without being forced into any predefined categories. In the coding process, the basic unit of analysis was defined as student statements and statement fragments that formed a meaningful whole. Codes with similar meanings were compared and grouped together to form draft themes. During the process of creating and organizing the codes, a coding framework was established by determining the name, scope, and relevant statements for each code. As the coding process progressed, newly emerging codes were compared with existing ones; codes with similar meanings were merged, while those with differing scopes were kept separate. This ensured that codes were distinguished from one another and that similar statements were consistently evaluated under the same code.

In the second stage, the core constructs comprising the quantitative dimension of the study (self-regulation, motivation, and attitude) were approached as a deductive framework. The resulting codes were linked to these constructs, and content directly related to them was further identified and organized under relevant subthemes. In this stage, the constructs previously defined for the quantitative dimension were not applied directly to the qualitative data as codes; rather, they were used to assess the relationship between the emerging codes and the study’s quantitative dimension.

To enhance the reliability and consistency of the qualitative analyses, the coding process was conducted not only by the principal investigator but also by an independent expert in Educational Sciences. Both experts independently coded the entire interview dataset. After the independent coding was completed, the two experts met to compare their codes and discuss any discrepancies. During the meeting, the interview data for each student were reviewed in turn; the code names, their scope, and their relationship to the relevant theme were evaluated together. Statements that had been coded differently were discussed, taking into account the student’s overall perspective and the statement’s context, and a consensus was reached on which code most accurately reflected the intended meaning. For example, when certain student statements could be associated with both the “self-regulation/time management” and “independent learning” categories, an assessment was made—taking into account the statement’s entirety and context—to determine which code more accurately represented the statement. In such cases of overlap, the scopes of the codes were compared, and care was taken to ensure that similar statements were consistently categorized under the same code. Furthermore, student statements that diverged from prevailing views on the implementation process or were negative in nature were not excluded from the analysis; instead, they were categorized under separate codes based on their content. For example, statements regarding resistance experienced at the beginning of the implementation, the cognitive load associated with the intensive use of questions, and fatigue resulting from active participation were not excluded simply because they did not align with positive views; rather, they were analyzed under the relevant codes.

The MAXQDA 20 software was used to organize, code, and visualize the interview data. Maps were created within the software to illustrate the relationships between codes. The thickness of the connecting lines on the maps indicates the strength of the relationship between the codes. These maps provided a clearer view of the codes that emerged from the qualitative data and their relationships. As a result of the analysis, the qualitative data were grouped under three main themes; the findings were supported by direct quotations; and participants were coded as K1, K2, and so on. In the qualitative analysis, the criteria of credibility, transferability, consistency, and verifiability were considered.

In line with the mixed-methods approach, to demonstrate the integration of qualitative findings with quantitative variables (data integration), qualitative codes were symbolically coded according to the quantitative variables with which they were primarily associated (symbol-coding). For codes that could not be directly linked, the existing representation was retained; the symbol and variable descriptions in the model map are presented in Table 3.

## 3. Results

### 3.1. Quantitative Results

#### 3.1.1. Results Regarding the Initial Equivalence of the Experimental and Control Groups

The mean pre-test and post-test scores for the SRS, MCAS, and MMS for the experimental and control groups are presented in Table 4.

As shown in Table 4, the descriptive statistics (means and standard deviations) for the pre-test and post-test scores on the Mathematics Attitude Scale, the Self-Regulation Scale, and the Mathematics Motivation Scale are presented. Furthermore, there is no significant difference between the groups in terms of pre-test scores on these scales (see Table 6).

A two-way mixed ANOVA was conducted to examine changes within groups as a function of time and group. Prior to the analysis, the homogeneity of variances was assessed using Levene’s test; this indicated that the Self-Regulation Scale (pre-test: *F*(1, 36) = 0.375, *p* = 0.544; post-test: *F*(1, 36) = 0.675, *p* = 0.417), the Mathematics Attitude Scale (pre-test: *F*(1, 36) = 0.233, *p* = 0.632; post-test: *F*(1, 36) = 0.229, *p* = 0.635) and the Mathematics Motivation Scale (pre-test: *F*(1, 36) = 1.188, *p* = 0.283; post-test: *F*(1, 36) = 0.056, *p* = 0.814) showed that the assumption was met for all measurements. Additionally, the homogeneity of the variance-covariance matrices was confirmed for self-regulation (Box’s M = 2.163, *F*(3, 381,693.991) = 0.677, *p* = 0.566), attitude (Box’s M = 3.831, *F*(3, 381,693.991) = 1.200, *p* = 0.308), and motivation (Box’s M = 3.773, *F*(3, 381,693.991) = 1.182, *p* = 0.315).

#### 3.1.2. Results on the Effect of the Experimental Intervention on Mathematics Motivation, Self-Regulation, and Attitudes Toward Mathematics (Mixed ANOVA)

**Figure 1:**
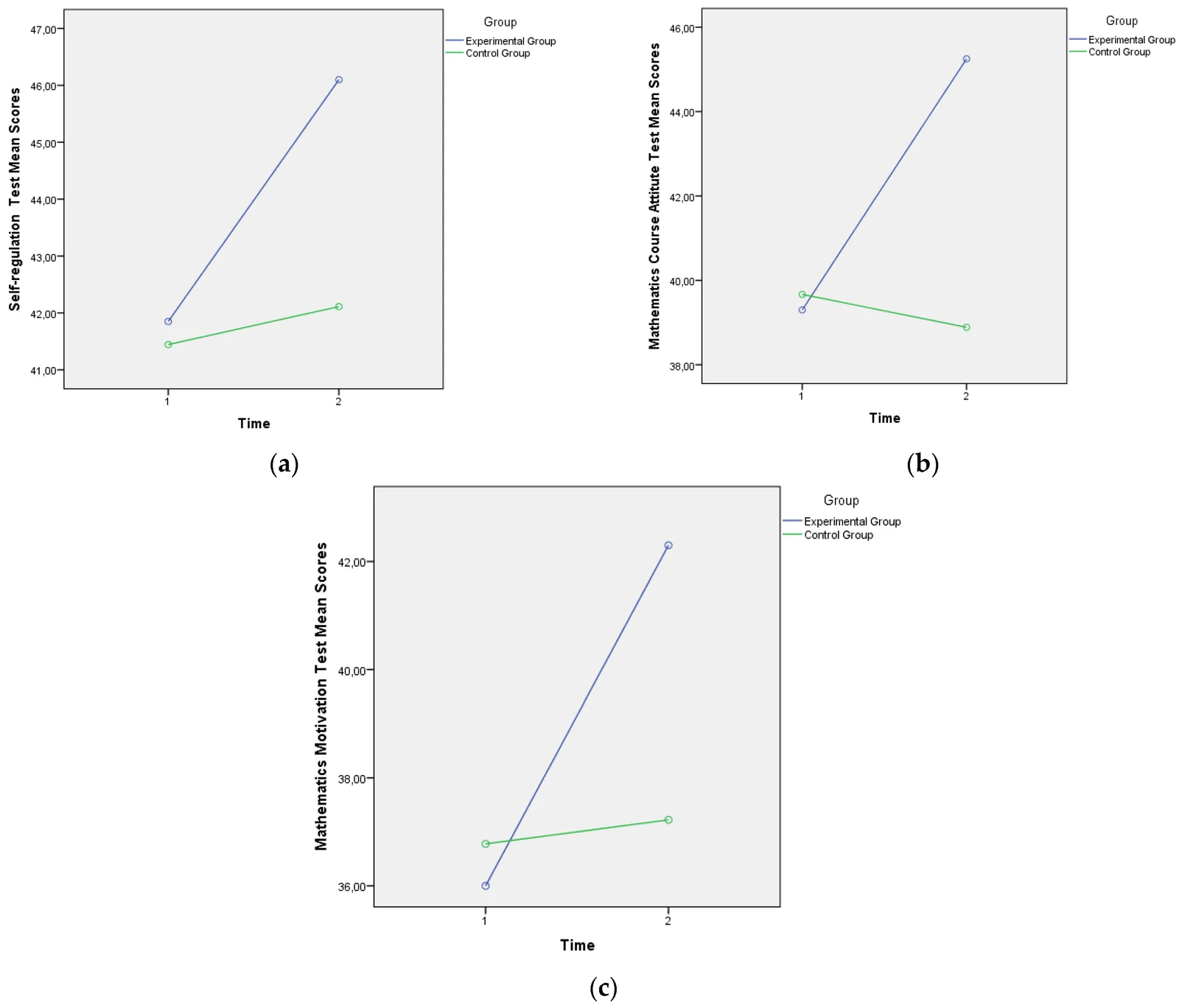
Time × Group interaction graphs for the self-regulation (**a**), attitude toward mathematics (**b**), and motivation in mathematics (**c**) scales.

A Two-Way Mixed ANOVA was conducted to examine the effect of the experimental procedure on Self-Regulation (SRSA), Attitude Toward Mathematics (MCAS), and mathematics Motivation (MMS) scores (Table 5). Graphs depicting the Time × Group interaction are presented in Figure 1, while the results of the Bonferroni post hoc comparisons between groups are presented in Table 5.

An examination of Table 5 reveals that the time × group interaction effect on students’ self-regulation scores was significant (*F*(1, 36) = 16.48, *p* < 0.05, *η_p_*^2^ = 0.314). The partial eta-squared value indicates that 31.4% of the variance in self-regulation scores was attributable to the experimental condition. According to the post hoc results (see Table 6), while no significant difference was found between groups in pre-test scores (Mean Difference = 0.406; *p* = 0.825 > 0.05), a significant difference in favor of the experimental group was determined in the post-test (Mean Difference = 3.989; *p* = 0.026 < 0.05). To further break down this interaction, tests of simple main effects revealed that the experimental group experienced a statistically significant increase from pre-test to post-test (*F*(1, 36) = 48.937, *p* < 0.001, *η_p_*^2^ = 0.576; Mean Difference = 4.250, *p* < 0.001), whereas the control group showed no significant change over time (*F*(1, 36) = 1.084, *p* = 0.305; Mean Difference = 0.667, *p* = 0.305). These findings indicate that self-regulation scores increased significantly over time in the experimental group, whereas no statistically significant change was observed in the control group, thereby supporting Hypothesis H1.

A significant time × group interaction effect was also found in mathematics attitude scores (*F*(1, 36) = 41.049, *p* < 0.001, *η_p_*^2^ = 0.533). The effect size indicates that a substantial portion—53.3%—of the variance in attitude scores is explained by the combined effect of the experimental intervention and time. Post hoc results showed that there was no significant difference in the pre-test (Mean Difference = −0.367; *p* = 0.912), but a significant difference in favor of the experimental group was found in the post-test (Mean Difference = 6.361; *p* = 0.046 < 0.05). To further break down this interaction, tests of simple main effects revealed that the experimental group experienced a statistically significant increase from pre-test to post-test (*F*(1, 36) = 67.781, *p* < 0.001, *η_p_*^2^ = 0.653; Mean Difference = −5.950, *p* < 0.001], whereas the control group showed no significant change over time (*F*(1, 36) = 1.042, *p* = 0.314; Mean Difference = 0.778, *p* = 0.314). To determine whether the results were influenced by the data distribution, a Mann–Whitney U test was also conducted using the post-test scores. The test confirmed a significant difference between the groups (U = 94.000, *p* = 0.012) and provided additional evidence of a statistically significant post-test difference. These findings indicate that mathematics attitude scores increased significantly over time in the experimental group, whereas no statistically significant change was observed in the control group, providing support for Hypothesis H2.

In the analyses of mathematics motivation scores, the time × group interaction effect was also significant (*F*(1, 36) = 43.207, *p* < 0.001, *η_p_*^2^ = 0.545). The partial eta-squared value indicates that 54.5% of the variance in mathematics motivation scores was associated with the time × group interaction. According to post hoc results, while no significant difference was found in pre-test scores (Mean Difference = −0.778; *p* = 0.655 > 0.05), a significant difference in favor of the experimental group was determined in the post-test (Mean Difference = 5.078; *p* = 0.008 < 0.05). To further break down this interaction, tests of simple main effects revealed that the experimental group experienced a statistically significant increase from pre-test to post-test (*F*(1, 36) = 105.588, *p* < 0.001, *η_p_*^2^ = 0.746; Mean Difference = −6.300, *p* < 0.001), whereas the control group showed no significant change over time (*F*(1, 36) = 0.473, *p* = 0.496; Mean Difference = −0.444, *p* = 0.496). This finding indicates that mathematics motivation scores increased significantly over time in the experimental group, whereas no statistically significant change was observed in the control group, providing support for Hypothesis H3.

### 3.2. Qualitative Results

**Figure 2:**
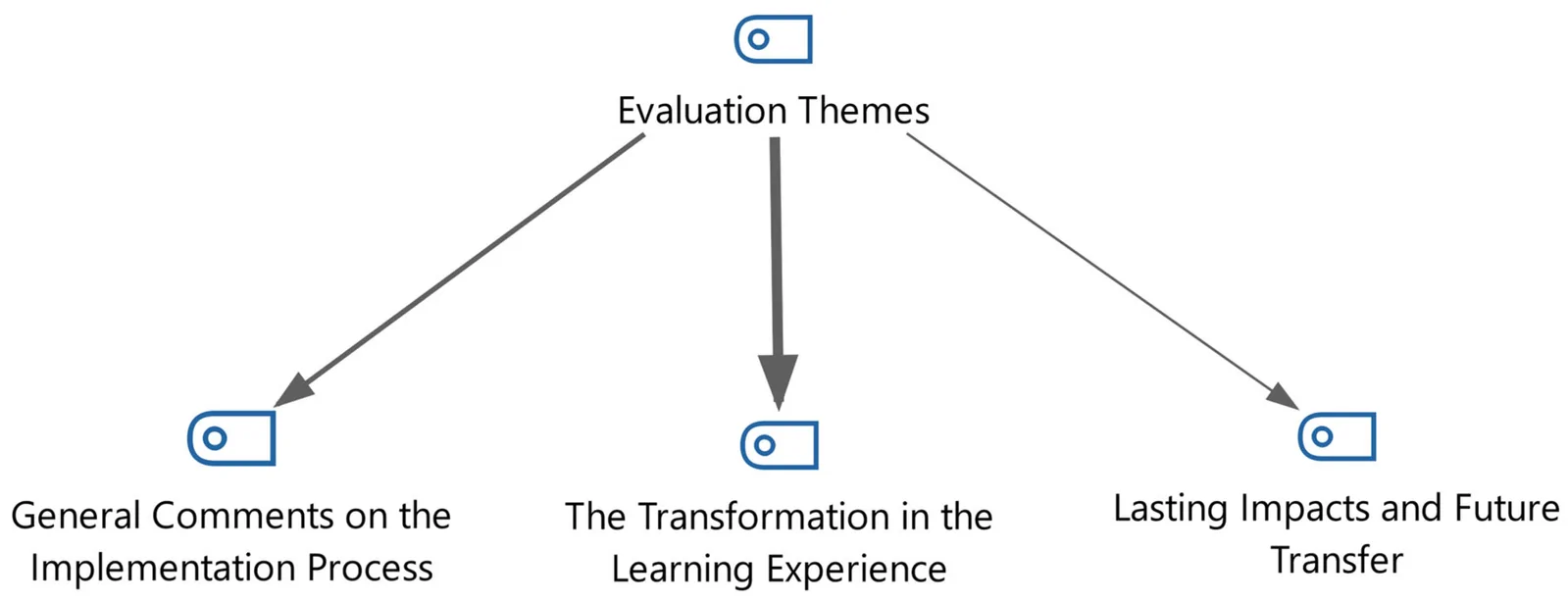
A map of the qualitative themes.

The findings from content analysis and data saturation were organized into three main themes. Participants’ evaluations of the duration, operation, and implementation process of the applied lesson sequence, as well as the system’s feasibility, constitute the first main theme. Changes in students’ attitudes toward math class, motivation, and perceptions of self-efficacy throughout the process, as well as in-class peer interactions, and participants’ perceptions of personal and interpersonal changes are grouped under the second main theme. Finally, participants’ reports on the use of self-regulation strategies in their general study habits and other disciplines following the experimental intervention formed the third main theme, focusing on participants’ perceptions of the potential continuation and transfer of strategy use. The model illustrating the main themes derived from the qualitative data is presented in Figure 2.

#### 3.2.1. Theme 1 (General Comments on the Implementation Process)

**Figure 3:**
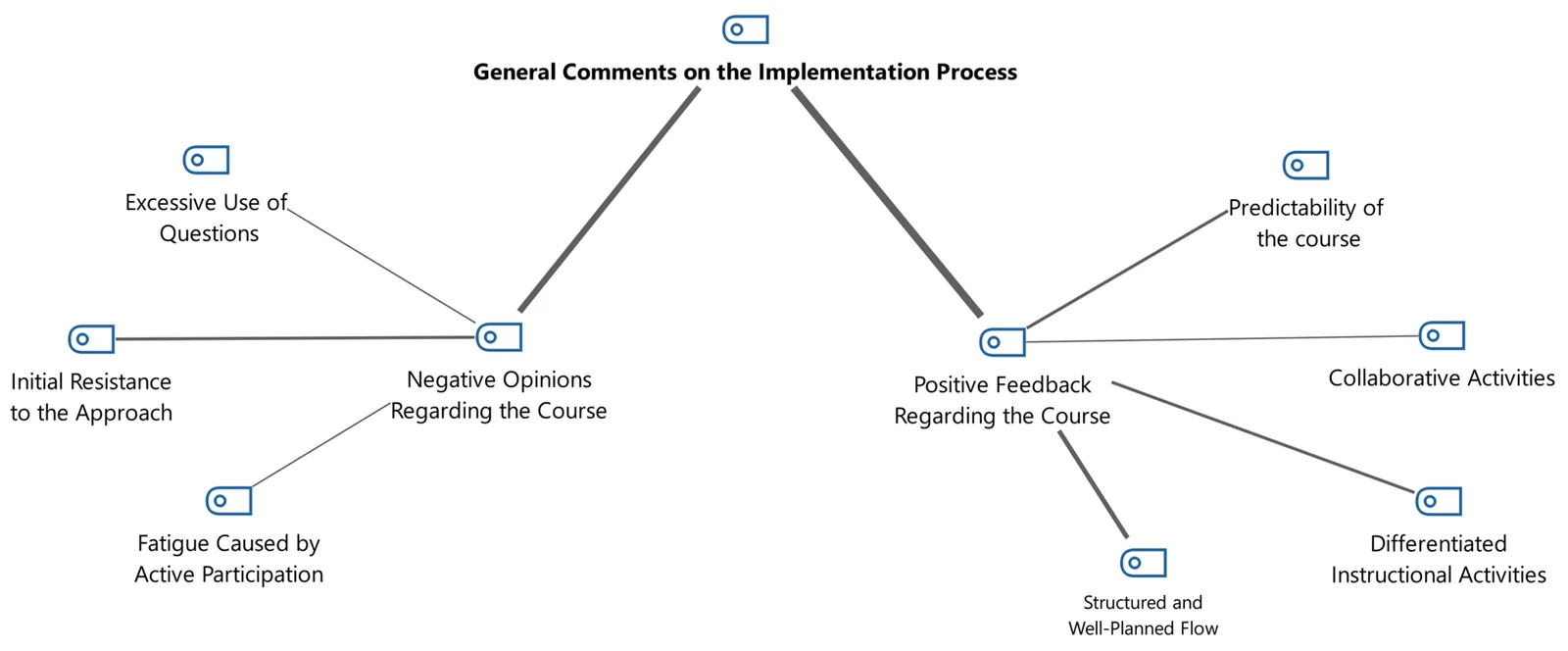
The map of subthemes and codes for general comments on the implementation process.

The first theme covers students’ views on the MLP-SRL application’s functionality and the process itself. The subthemes, codes, and conceptual organization associated with this theme are presented in the model map shown in Figure 3.

As shown in Figure 3, the qualitative findings regarding the application process are organized under two main subthemes: “Positive Feedback Regarding the Course” and “Negative Opinions Regarding the System.” Students evaluated the course structure and instructional activities positively. Regarding the predictability of the course, having the objectives and activities shared at the beginning of class put them at ease. Student 6 described this situation, saying, “It really put me at ease when the teacher clearly told us at the beginning of class what we would be learning and doing today. We knew in advance what we would be doing in class.” Structured class routines helped them maintain their focus. Inquiries based on incorrect equations made the lesson more engaging. Student 4 described this experience, saying, “The parts where the teacher would intentionally bring incorrectly solved equations to the board or in front of us and say, ‘Let’s find the mistake here,’ were great. It felt very fun and dynamic to me.” Furthermore, working with peers made the learning process more enjoyable.

The “Negative Perceptions” subtheme reflects the difficulties students experienced while adapting to the new learning structure. Students noted that the transition to a structure different from their familiar teaching approach was challenging at first. Reflective questions sometimes created a cognitive load. Student 3 explained, “Constantly thinking about those questions at the beginning and end of class and trying to answer them for the teacher was sometimes exhausting. We were answering way too many questions.” Being constantly active required greater mental effort. Student 7 described this situation, saying, “In a traditional class, we were comfortable just leaning back and listening to the instructor. Here, having to be active from the beginning to the end of class wore me out.

#### 3.2.2. Theme 2 (The Transformation in the Learning Experience)

**Figure 4:**
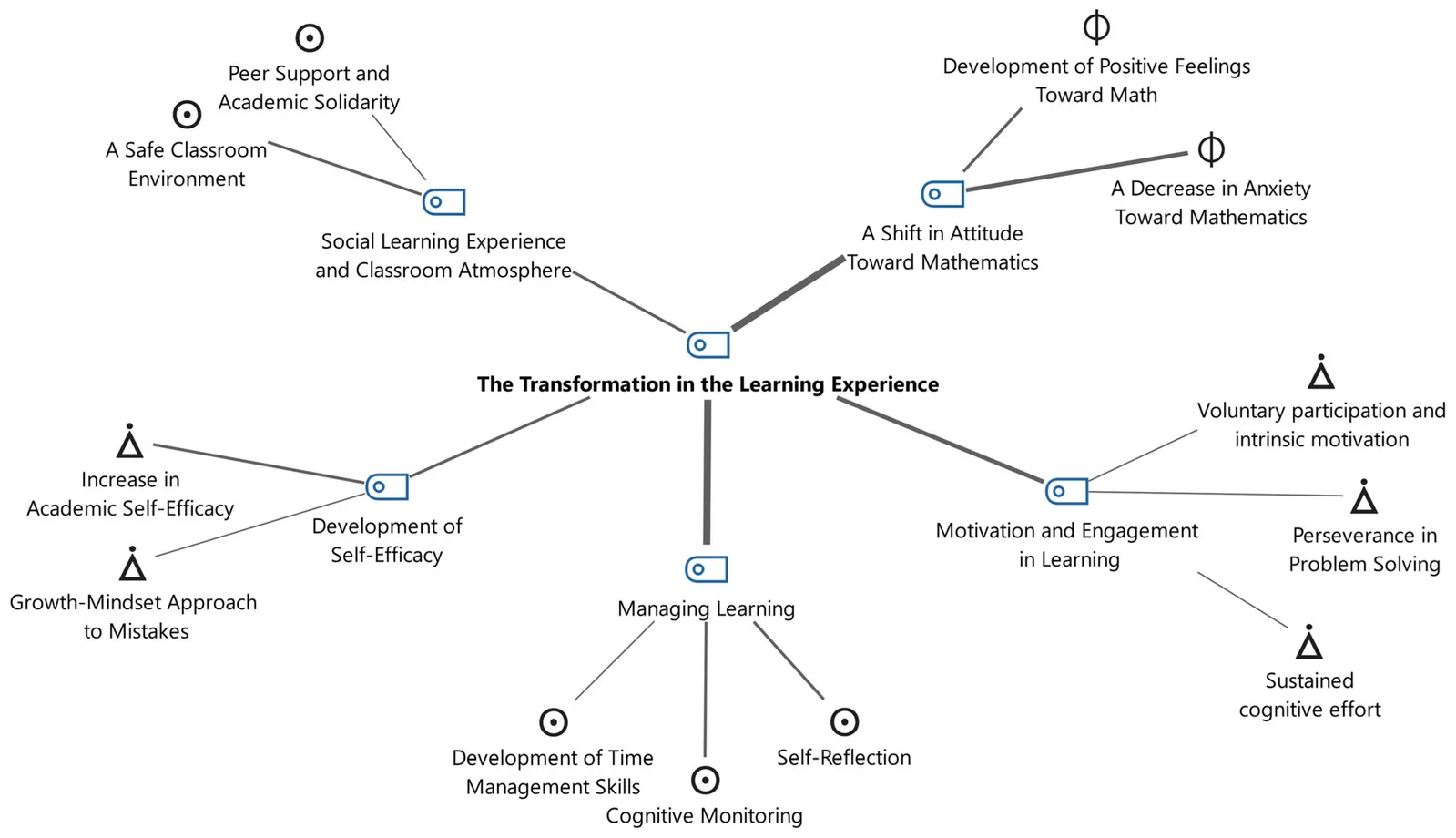
A map of the subthemes and codes for transformation in the learning experience.

This theme, which is the main focus of the qualitative findings, addresses the perceptual changes students experienced across the internal, affective, and relational dimensions during the experimental intervention. In line with the mixed-methods approach, the findings were evaluated in relation to the variables of Self-Regulation, Mathematics Motivation, and Attitude Toward Mathematics. A map showing the subthemes, codes, and associated quantitative variables is presented in Figure 4.

Upon examining the conceptual map in Figure 4, the qualitative findings regarding students’ perceived changes in the learning experience are grouped under five subthemes. The “Managing Learning” subtheme reflects students’ perceptions of changes in metacognitive control processes throughout the process. Students reported becoming more aware of their learning processes. Under the “cognitive monitoring” code, they reported becoming more aware of what they did and did not understand; under the “development of time management skills” code, they reported beginning to make more realistic plans. Student 1 explained, “At first, I misjudged the time it would take me to finish the questions. Over time, I learned to set more realistic time limits for myself.”

The second subtheme, “A Shift in Attitude Toward Mathematics,” reflects students’ perceptions of affective changes regarding mathematics and is linked to their attitude toward mathematics. Regarding the “Decrease in Anxiety Toward Mathematics” code, students indicated that their negative perceptions had changed; Student 6 described this transformation as follows: “I used to get stressed out just thinking about math class starting; I thought I wouldn’t be able to do it. When we did error-finding puzzles and things like that in these classes, that prejudice of mine was broken, and the class doesn’t seem so scary anymore.” Similarly, within the “Development of Positive Feelings Toward Math” category, students reported experiencing more positive feelings.

The codes falling under the third subtheme, “Motivation and Engagement in Learning,” and the fourth subtheme, “Development of Self-Efficacy,” were closely associated with students’ motivation in mathematics. Within the third subtheme, codes such as perseverance in problem-solving, sustained cognitive effort, voluntary participation, and intrinsic motivation reflected students’ reported perceptions of changes in their engagement. For instance, Student 5 explained the change in her determination with the following statement: “In the past, when I struggled with a problem, I would give up right away. Now, when I can’t do it, I try a little harder.” Similarly, the fourth subtheme, “Development of Self-Efficacy,” reflected students’ evolving beliefs in their ability to succeed and to overcome challenges through planning and the use of strategies. Rather than viewing mistakes as failures, students reported adopting a growth-oriented approach, perceiving errors as a natural part of learning. As Student 1 shared: “I used to get discouraged when I made a mistake. Now, by figuring out where I went wrong, I’ve realized that making mistakes is actually a normal part of learning the subject, so I don’t give up.”

The fifth subtheme, “Social Learning Experience and Classroom Atmosphere,” reflects students’ processes of managing the learning environment and is linked to self-regulation. Under the code “Peer Support and Academic Solidarity”, students noted that the processes of seeking help and working together had become easier. Under the code “Safe Classroom Environment”, students noted that they perceived the learning environment as more comfortable, with greater emphasis on mutual support than competition. Student 3 shared the following view: “Everyone was trying to help each other, and since no one made fun of anyone else when they made a mistake, the classroom environment was very comforting.”

#### 3.2.3. Theme 3 (Lasting Impacts and Future Transfer)

**Figure 5:**
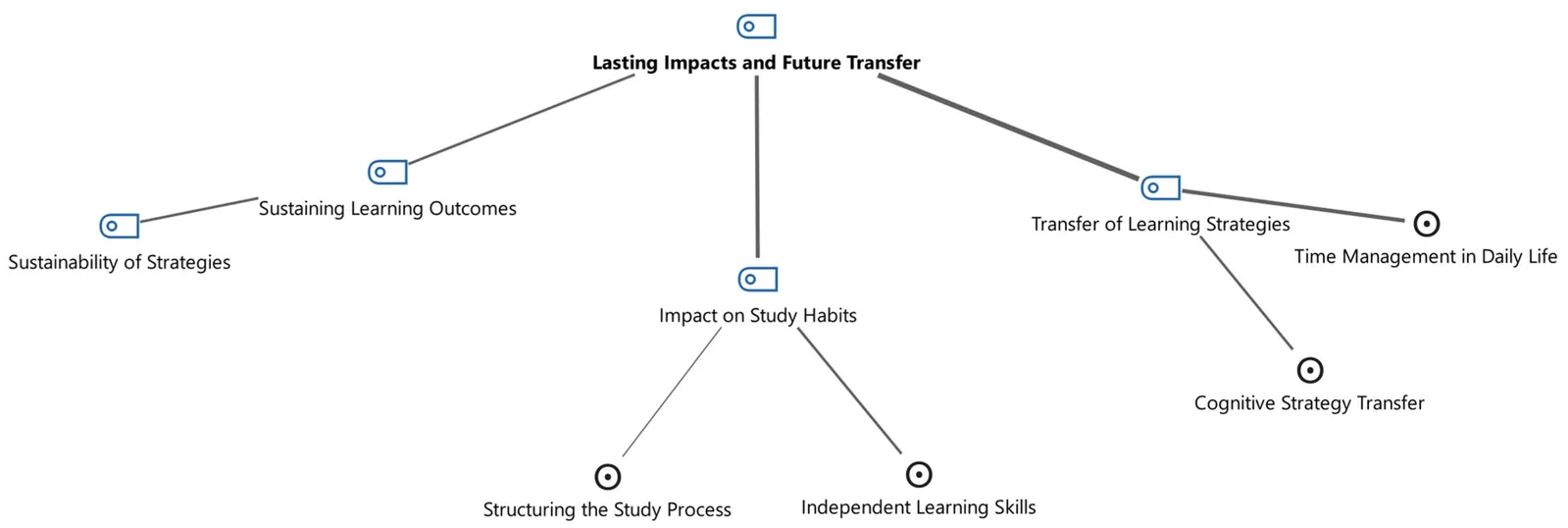
A map of the subthemes and codes for lasting impacts and future transfer.

The final main theme of this study examines students’ reports regarding how the strategies used during the MLP-SRL application process were perceived to be reflected in their academic routines, other academic contexts, and daily life after the program. In this context, students’ reports concerning the use of cognitive and metacognitive strategies across different contexts, along with related quantitative variables, were examined. A symbolic representation showing the subthemes, codes, and relationship patterns is presented in Figure 5.

As shown in Figure 5, findings on students’ perceptions of the potential continuation of strategy use and its transfer to other contexts are grouped into three subthemes. The “Impact on Study Habits” subtheme reflects students’ reports of changes in how they organize their independent study processes and is associated with self-regulation. Under the “Structuring the Study Process” code, students indicated that they applied time management and goal-setting strategies to their homework. Student 3 described this situation as follows: “After this system, when I sit down at my desk at home, I started doing the same things I do at school. First, I set a time limit for myself and plan what I’m going to work on…” Under the Independent Learning Skills category, students indicated that they could manage their learning processes without external guidance.

The subtheme “Transfer of Learning Strategies” reflects students’ reports regarding the use of self-regulation strategies across different subjects and daily life. In the category of cognitive strategy transfer, students reported applying the habits of goal setting and error analysis they developed in math to their science classes. Student 7 explained this situation, saying, “I’m now applying that logic of setting goals and looking for errors that we used in math to my Physics and Chemistry studies as well.” Under the “Time Management in Daily Life” code, students noted that their time-planning skills were also reflected in their routines outside of class (Student 6: “Thinking about how much time I should allocate to each task when planning my day has become a habit for me.”).

The subtheme “Sustaining Learning Outcomes” encompasses students’ views regarding the potential continuation of strategy use following the implementation process. Under the “Sustainability of Strategies” code, students indicated they would continue to use the planning and self-regulation skills they reported using during the MLP-SRL implementation. Student 1 expressed this by saying, “I definitely won’t give up this method while preparing for the college entrance exam next year; I’ll study entirely according to this plan.”

## 4. Discussion

In this study, quantitative and qualitative data were integrated during the interpretation phase. While the quantitative findings indicated significant changes in students’ self-regulation, attitudes toward mathematics, and motivation over time in the experimental group, the qualitative findings provided insight into how students perceived and interpreted these changes and reported using related strategies in other academic contexts.

The findings indicated that students’ self-regulation scores, mathematics attitudes, and mathematics motivation changed significantly over time in the experimental group, supporting the corresponding hypotheses. This is consistent with the view that self-regulation is not a fixed trait but a competency that can be developed through structured instruction. Qualitative data were also consistent with the quantitative findings, as students reported using goal setting, cognitive modeling, error analysis, and strategic study skills across disciplines. Consistent with Zimmerman’s cyclical stage model, these findings support the interpretation that structured instructional processes may facilitate the use of domain-specific cognitive strategies, along with metacognitive monitoring and positive affective processes.

The first hypothesis of the study (H1) predicted that the experimental group, which received instruction using MLP-SRL, would show a greater increase in self-regulation scores compared to the control group. The significant time × group interaction and the greater post-test scores of the experimental group, despite equivalent pre-test scores, supported H1 and indicated a pattern of change in self-regulation scores consistent with the expected contribution of MLP-SRL. When the quantitative and qualitative findings are considered together, the increase in self-regulation scores is further supported by the “Managing Learning” subtheme identified in the qualitative data. The students’ reported changes in self-reflection, cognitive monitoring, time management, and their ability to recognize and address their own shortcomings suggest that the structured lesson flow may have supported the coordinated use of cognitive strategies and metacognitive regulation. These findings further suggest that integrating cognitive strategy use with metacognitive monitoring may help students become more aware of how they approach mathematics tasks and regulate their learning accordingly. This is particularly relevant to mathematics learning, where students need to coordinate strategic thinking, monitoring, and evaluation when working with complex and abstract problems. This interpretation aligns with Zimmerman’s cyclical model, in which instructional support for forethought, performance, and self-reflection can foster students’ active regulation of their learning (Zimmerman, 2000). The current findings are also consistent with research showing that strategy-based interventions can enhance self-regulation, although the magnitude of these effects may vary according to program characteristics, teacher practices, and the dimensions examined (Heirweg et al., 2021; Schuler et al., 2024; Sins et al., 2023). In this context, evidence that structured SRL practices support self-assessment and strategic cognitive management (Kersna et al., 2025) is consistent with studies indicating that self-regulatory mechanisms can be systematically developed through explicit and strategy-focused instruction (Alvi & Gillies, 2021; Greenquist-Marlett et al., 2025; Sins et al., 2023). Overall, the findings are consistent with the view that self-regulation is a malleable competency that may be strengthened through appropriately designed instructional interventions.

The second hypothesis of the study (H2) predicted that the combined effect of the experimental intervention and time would increase students’ attitude scores toward mathematics, and the analysis results supported H2. The fact that the groups, which were equivalent on the pre-test, showed a significant difference in favor of the experimental group on the post-test indicates a pattern of change in students’ affective attitudes toward mathematics that was consistent with the expected contribution of the intervention. The mixed-methods findings demonstrated that the quantitative increase in attitudes aligned with the qualitative subtheme “A Shift in Attitude Toward Mathematics.” Students’ reports of reduced math anxiety, changes in their “I can’t do it” perceptions, and the development of positive emotions and interest were consistent with this attitudinal change, although these qualitative perceptions should not be interpreted as direct evidence of measured reductions in anxiety. In particular, the integration of cognitive strategies and metacognitive monitoring may have helped students approach mathematics tasks more strategically, monitor their progress, and develop greater confidence in managing difficulties. The findings are consistent with the literature demonstrating the relationship between self-regulation skills and positive attitudes toward mathematics (Ocak & Yamaç, 2013; Tee et al., 2018; Khizar et al., 2024). However, given the predominance of correlational studies in the literature, this study—which experimentally examines the effect of self-regulation on attitude and integrates it with qualitative data—provides evidence that helps address this gap (Clyde et al., 2022; Hıdıroğlu & Hıdıroğlu, 2026). Studies showing that self-regulation strategies enhance perceptions of confidence, motivation, and efficacy also support the present findings (Tee et al., 2018; Ocak & Yamaç, 2013; Khizar et al., 2024). In conclusion, the observed increase in students’ perceived control over their learning processes was accompanied by more positive attitudes, and the mixed-methods findings support the relevance of systematically integrating self-regulated learning processes into mathematics instruction to address negative beliefs.

The third hypothesis of the study (H3) predicted that the model would increase student motivation, and the analyses supported this hypothesis. The significant difference in favor of the experimental group on the final test indicates a pattern of change in academic motivation consistent with the expected contribution of the self-regulation instruction. The mixed-methods findings showed that the quantitative increase in motivation was consistent with the qualitative subthemes of “Desire and Engagement in Learning” and “Self-Efficacy Development.” Students’ reports of persistence in problem-solving, sustained cognitive effort, and strengthened self-efficacy beliefs were consistent with this motivational change, while process-oriented monitoring may have supported these experiences. These findings are consistent with studies showing that self-regulation skills and motivational beliefs develop in tandem (Cleary & Chen, 2009; ElAdl & Alkharusi, 2020). Research indicating that structured interventions increase perseverance, task value, and effort (Granello et al., 2025; Léon et al., 2015) and that self-efficacy and mastery goals support cognitive processes (Tee et al., 2020) supports the present findings. On the other hand, it is also known that the effects of self-regulation interventions on motivational outcomes may vary or emerge through indirect mechanisms (Gabriel et al., 2020; Rovan et al., 2023). The explicit and systematic instruction used in this study may have supported students’ perceptions of control and their engagement with learning. Consistent with Pintrich (1999) and Wolters (2003), students attributed their success to effort and strategy use rather than ability. In line with studies indicating that motivational regulation and strategy use exhibit similar patterns across different domains (Pintrich & De Groot, 1990; Wolters & Pintrich, 1998; Guo et al., 2021), the current findings provide support for the relevance of self-regulation training as a framework for supporting intrinsic motivation, task value, and academic self-efficacy beliefs.

Students’ views on the MLP-SRL process revealed both the facilitating aspects of the structured teaching plan and the challenges they faced in adapting to it. While sharing objectives at the beginning of the lesson provided predictability, inquiries based on incorrect equations and collaborative activities made the process engaging. In particular, deliberate mathematics error analysis encouraged students to articulate their reasoning and view errors as opportunities for learning rather than as sources of failure. These findings were consistent with the view that structured SRL routines may support a more positive classroom climate and were aligned with Zimmerman’s (2002) “anticipation” phase. On the other hand, initial resistance, the cognitive load posed by reflective questions, and the requirement for continuous active participation highlight the difficulties of transitioning from traditional to self-regulated learning. Studies showing that self-regulation interventions may initially create a cognitive load and that strategy use develops over time are consistent with these findings (Léon et al., 2015; Wolters, 2003). The reported adaptation as the process progressed may indicate increasing familiarity with the instructional routines, while students’ reports of motivational and cognitive regulation were consistent with the process of developing greater self-regulation (Pintrich, 1999). In-process guidance may also have supported students in approaching difficulties as learning opportunities.

Student evaluations suggest that the MLP-SRL program may support students’ perceived development of transferable learning strategies. Students’ reports of managing their learning processes at home without external guidance suggest perceived development of greater independence in learning (Zimmerman, 2002). Similarly, students’ self-reported use or intended use of goal setting and error analysis in other disciplines, such as physics and chemistry, suggests perceived interdisciplinary transfer of metacognitive strategies. However, these findings reflect students’ self-reported experiences and intentions rather than directly observed or longitudinally demonstrated transfer or behavioral persistence. The literature showing that self-regulation interventions can support strategic learning, self-efficacy, and learning behaviors across different domains provides a theoretical basis for interpreting these findings (Elhusseini et al., 2022; Lai et al., 2018; Schraw et al., 2006). Likewise, students’ intentions to use these strategies in future exam preparation indicate perceived potential for continued use, rather than demonstrating the intervention’s long-term or cross-disciplinary impact.

### Limitations and Future Research

This study has several limitations. First, because the intervention was conducted at a single high school, the generalizability of the findings to diverse school contexts and socioeconomic backgrounds is limited. Second, although the 8-week intervention was associated with significant gains, longitudinal studies with follow-up measurements are needed to assess whether these cognitive and metacognitive outcomes are sustained over time. Third, this study did not include an objective measure of mathematics achievement or problem-solving performance. Although students’ experiences related to problem-solving were explored through qualitative interviews, these findings should not be interpreted as evidence of improved objective mathematics performance. Future research should incorporate objective measures of mathematics achievement alongside self-regulation, motivation, and attitude measures. Fourth, this study focused exclusively on 11th-grade mathematics. Future research should examine the applicability and strategy transfer of the MLP-SRL framework across different grade levels and STEM disciplines, and investigate the role of teacher scaffolding and instructional support in diverse learning environments.

## 5. Conclusions

The results indicate that self-regulation capacity, positive attitudes toward mathematics, and mathematics motivation are dynamic and amenable to change within a structured instructional context. The MLP-SRL-based lesson plan was associated with changes in students’ self-regulation, attitudes toward mathematics, and motivation. These findings highlight the importance of integrating cognitive and metacognitive strategies into self-regulated mathematics instruction. In particular, providing structured opportunities for goal setting, strategy use, metacognitive monitoring, error analysis, and self-reflection can help students manage their learning processes more effectively while working on complex mathematics tasks.

The results also highlight the importance of teacher guidance, particularly in the early stages, as students adapt to self-regulated learning. Providing appropriate guidance when students experience cognitive load or resistance can help them develop control over their learning over time and recognize the role of effort and strategy in their learning processes. Consequently, the findings suggest that mathematics instruction may benefit from going beyond content delivery by providing structured opportunities for students to plan, monitor, evaluate, and reflect on their learning. Overall, the findings highlight the importance of addressing cognitive, metacognitive, and affective dimensions together in self-regulated mathematics instruction and suggest that structured MLP-SRL-based instruction may support students’ development across these dimensions within the mathematics learning context.

## Figures and Tables

**Table 1:** Intervention design applied in study.

Method	Group	Research Dimension	Pre-Intervention (Pre-Tests)	Experimental Intervention	Post-Intervention (Post-Tests)
Mixed	Experimental Group	Quantitative	SRSA/MCAS/MMS	MLP-SRL	SRSA/MCAS/MMS
		Qualitative	-		SSI
	Control Group	Quantitative	SRSA/MCAS/MMS	CC	SRSA/MCAS/MMS

Note. SRSA = Self-Regulation Scale for Adolescents; MCAS = Mathematics Course Attitude Scale; MMS = Mathematics Motivation Scale; SSI = Semi-Structured Interview; MLP-SRL = Mathematics Lesson Plan Based on Self-Regulated Learning; CC = Current Curriculum.

**Table 2:** Shapiro–Wilk normality test results of students’ pre-test and post-test scores.

Variable	Group	Time	Shapiro–Wilk	*df*	*p*	Kurtosis	Skewness
Self-Regulation Scale (SRSA)	Experimental	Pre-test	0.947	20	0.322	0.092	0.471
		Post-test	0.941	20	0.255	−1.050	0.238
	Control	Pre-test	0.963	18	0.655	−0.025	0.517
		Post-test	0.952	18	0.465	0.630	0.541
Mathematics Course Attitude Scale (MCAS)	Experimental	Pre-test	0.892	20	0.029	1.020	1.108
		Post-test	0.860	20	0.012	1.540	1.048
	Control	Pre-test	0.884	18	0.021	1.199	1.331
		Post-test	0.853	18	0.009	0.363	1.174
Mathematics Motivation Scale (MMS)	Experimental	Pre-test	0.956	20	0.474	−0.237	−0.448
		Post-test	0.970	20	0.748	0.132	0.235
	Control	Pre-test	0.951	18	0.447	−1.202	0.027
		Post-test	0.952	18	0.464	0.183	−0.724

**Table 3:** Symbolic mapping of qualitative codes to quantitative variables.

Map Geometric Symbol	Primary Variable Represented	Reason for Selecting the Symbol	Code Display on the Map (Example)
⨀	Self-Regulation	Symbolizes the student’s focus on their own mental processes and internal monitoring.	⨀ Time Management and Planning Stress
⧊	Mathematics Motivation	Symbolizes cognitive effort during the learning process and the upward momentum of motivation.	⧊ Persistence and Cognitive Effort Toward a Solution
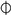	Attitude Toward Mathematics	Symbolizes the transition from fears about the subject to a phase of positive perception.	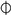 Overcoming Fear of Mathematics

**Table 4:** Arithmetic means and standard deviations of the pre-test and post-test scores on the self-regulation scale, mathematics attitude scale, and mathematics motivation scales for the experimental and control groups.

Variable	Group	Pre-Test Mean	*SD*	Post-Test Mean	*SD*
Self-Regulation Scale (SRSA)	Experimental	41.85	6.10	46.10	5.45
	Control	41.44	4.98	42.11	5.10
Mathematics Course Attitude Scale (MCAS)	Experimental	39.30	10.80	45.25	9.30
	Control	39.66	9.23	38.88	9.64
Mathematics Motivation Scale (MMS)	Experimental	36.00	4.87	42.30	5.96
	Control	36.77	5.75	37.22	5.18

**Table 5:** Results of a two-factor mixed-design analysis of variance (mixed ANOVA) on self-regulation, attitudes toward mathematics, and motivation in mathematics scores.

Variable	Source of Variance	Sum of Squares	*df*	Mean Square	*F*	*p*	$\eta_{p}^{2}$
Self-Regulation Scale (SRSA)	**Within-group effects**						
	Time	114.507	1	114.507	31.023	<0.001	0.463
	Time × Group	60.822	1	60.822	16.479	<0.001	0.314
	Error (Time)	132.875	36	3.691			
	**Between-group effects**						
	Group	91.474	1	91.474	1.640	0.208	0.044
	Error	2007.697	36	55.769			
Mathematics Course Attitude Scale (MCAS)	**Within-group effects**						
	Time	126.719	1	126.719	24.261	<0.001	0.403
	Time × Group	214.404	1	214.404	41.049	<0.001	0.533
	Error (Time)	188.031	36	5.223			
	**Between-group effects**						
	Group	170.211	1	170.211	0.914	0.346	0.025
	Error	6707.697	36	186.325			
Mathematics Motivation Scale (MMS)	**Within-group effects**						
	Time	215.467	1	215.467	57.321	<0.001	0.614
	Time × Group	162.415	1	162.415	43.207	<0.001	0.545
	Error (Time)	135.322	36	3.759			
	**Between-group effects**						
	Group	87.584	1	87.584	1.566	0.219	0.042
	Error	2013.100	36	55.919			

Note. $\eta_{p}^{2}$ = Partial eta-squared (effect size); *df* = degrees of freedom; *F* = ANOVA test statistic.

**Table 6:** Results of post hoc comparisons between groups regarding self-regulation, attitudes toward mathematics, and motivation in mathematics scores.

Variable	Time	(I) Group	(J) Group	Mean Difference (I–J)	*SE*	*p*	95% Confidence Interval	
							Lower Limit	Upper Limit
Self-Regulation Scale (SRSA)	Pre-test	Experimental	Control	0.406	1.821	0.825	−3.288	4.099
	Post-test	Experimental	Control	3.989 *	1.721	0.026	0.500	7.478
Mathematics Course Attitude Scale (MCAS)	Pre-test	Experimental	Control	−0.367	3.280	0.912	−7.020	6.286
	Post-test	Experimental	Control	6.361 *	3.075	0.046	0.124	12.598
Mathematics Motivation Scale (MMS)	Pre-test	Experimental	Control	−0.778	1.725	0.655	−4.277	2.721
	Post-test	Experimental	Control	5.078 *	1.823	0.008	1.381	8.775

Note. Correction for multiple comparisons: Bonferroni * *p* < 0.05.

## Data Availability

The datasets generated and analyzed in the current study are available from the corresponding author upon reasonable request, subject to privacy and ethical restrictions.

## Appendix A. Mathematics Lesson Plan Based on Self-Regulated Learning with Integrated Cognitive and Metacognitive Strategies (MLP-SRL)

**FORETHOUGHT PHASE**				
**Sub-Phase**	**Sub-Dimension**	**Activity/Question (Step No.)**	**Strategy Used**	**Description/Mathematics Application**
		**1. Greeting and cognitive preparation**	Affective & Motivational Strategies	Managing the classroom atmosphere, helping students focus on abstract mathematics thinking.
		**2. Sharing the mathematics topic and goals with students**	Metacognitive Strategies (Planning/Goal Setting)	Sharing the day’s learning objectives and target problem types.
		**3. Students organizing their own learning environment**	Resource Management Strategies (Organizing the study environment)	Preparing math lesson materials and removing distractions.
		**4. Checking and reinforcing prior learning outcomes**	Cognitive Strategies (Rehearsal & Retrieval)	Quickly reviewing previous formulas or theorems that serve as the foundation for the new topic.
		**5. Asking topic-oriented self-regulation questions:**		
Task Analysis	a. Goal Setting	*Why? Why should you learn this mathematics topic?*	Metacognitive Strategies (Planning)	Emphasizing the topic’s relevance to daily life or mathematics enhances task value and engagement in the lesson.
		*What? What can you learn about this mathematics topic?*	Metacognitive Strategies (Planning)	Having students set cognitive goals related to the specific mathematics skills to be acquired in that lesson (e.g., deriving formulas, problem-solving).
	b. Strategic Planning	*How? Which mathematics method/strategy (e.g., drawing a diagram, algebraic substitution, trial-and-error) is the best way for you to learn this topic?*	Metacognitive Strategies (Planning)/Resource Management	Having students develop a solution plan by selecting in advance the mathematics method they will use (e.g., drawing diagrams, algebraic simplification, etc.).
		*Where? How will you organize your study space and deal with distractions?*	Metacognitive Strategies (Planning)/Resource Management	Identifying external factors that could disrupt concentration and math anxiety, and taking preventive measures to maintain focus.
		*With Whom? Who should you seek help from if you struggle with this mathematics topic?*	Resource Management Strategies (Help-Seeking)	Developing a plan to seek academic help—such as peer collaboration or teacher support—when encountering difficulties in mathematics operations.
		*When? How much time should you allocate to studying and practicing this mathematics topic?*	Metacognitive Strategies (Planning)	Having the student plan the in-class independent problem-solving process within a time-bound framework based on the topic’s difficulty level.
Self-Motivational Beliefs	Self-Efficacy	*Do you feel ready to learn and apply this topic in mathematics?*	Metacognitive Strategies (Monitoring)/Critical Thinking	Examining students’ perceptions of self-efficacy and their readiness to tackle complex mathematics tasks.
	Outcome Expectations	*Where will you use what you have learned in mathematics today, and how will it be useful to you?*	Metacognitive Strategies (Planning)	Raising awareness of how mathematics reasoning contributes to future academic success and problem-solving skills.
	Task Valuing or Intrinsic Interest	*Why might this mathematics topic be important for your development?*	Metacognitive Strategies (Planning)/Critical Thinking	Fostering intrinsic motivation in students—beyond test score anxiety—toward mathematics discovery and logical reasoning.
	Goal Orientation	*What could be your main purpose of learning this mathematics topic?*	Metacognitive Strategies (Planning)	Encouraging students to move away from a rote-learning approach and focus on deeply understanding mathematics relationships (learning orientation).

**PERFORMANCE AND VOLITIONAL CONTROL PHASE**				
**Sub-Phase**	**Sub-Dimension**	**Activity/Question (Step No.)**	**Strategy Used**	**Description/Mathematics Application**
		**6. Lesson Implementation:**		
		Formula Deconstruction & Active Questioning	Cognitive Strategies (Rehearsal and Elaboration)	Breaking down complex formulas and theorems into their components, and understanding the logical steps through guiding questions
		Step-by-Step Mathematics Modeling & Execution	Cognitive Strategies (Rehearsal, Elaboration, and Organizational Strategies)	Analyzing the “given-required” structure in problem-solving, transforming data into algebraic models, and organizing the steps of the process
		Graphical and Geometric Representation Analysis	Cognitive Strategies (Elaboration & Organization)	The concretization of abstract mathematics concepts through dynamic software, graphics, or geometric visualization tools
		Real-World Math Modeling (Contextual Problems)	Critical Thinking	Designing contextual problems based on real-life scenarios and translating verbal situations into mathematics language.
		Mathematics Error Analysis Challenge	Cognitive Strategies (Organization)/Critical Thinking	Presenting students with intentionally incorrectly solved equations to help them identify logical and computational errors; preventing conceptual misunderstandings.
		Collaborative Peer Problem-Solving Puzzles	Resource Management (Peer Learning)/Critical Thinking	Developing skills in peer collaboration, effort regulation, and seeking academic support through challenging math activities.
**SELF-REFLECTION PHASE**				
		**7. End-of-Topic Evaluation Questions**	Metacognitive Strategies (Evaluation)	Conducting brief formative assessments to measure mastery of daily math objectives
		**8. Asking topic-oriented self-regulation questions:**		
		*Why? Why was it important for us to learn this mathematics topic?*	Metacognitive Strategies (Evaluation)	Fostering a positive attitude toward the subject by encouraging students to question the underlying rationale of mathematics proofs or logical reasoning steps.
		*What? What did you learn today, and how/where can you use this mathematics knowledge?*	Metacognitive Strategies (Evaluation)	Helping students recognize how the mathematics theorems or relationships they have learned can be applied to different types of problems.
		*How? What did you do when you struggled during problem-solving, and how should you study next time?*	Metacognitive Strategies (Monitoring)/Resource Management (Effort Regulation)	Enabling students to analyze how they overcome cognitive barriers encountered in complex problems and to revise their cognitive strategies for future tasks.
		*Where Did you manage your study environment and cope with any distractions during the mathematics tasks?*	Resource Management (Organizing the Time & Study Environment)	Assessing students’ success in managing distractions that disrupt focus during mathematics tasks and in controlling math anxiety.
		*With Whom? Did you seek support from a peer or teacher when you got stuck on a math step?*	Resource Management (Help-Seeking)	Evaluating students’ behavior in seeking effective academic help and utilizing social resources when they encounter difficulties in mathematics tasks.
		*When? Did you manage your time well during independent mathematics practice?*	Resource Management (Organizing the Time & Study Environment)	Examining students’ ability to use time efficiently during in-class problem-solving and application processes.
Self-Judgment	a. Self-Evaluation	*How do you verify that you have mastered this math topic? What are your strengths and remaining gaps?*	Metacognitive Strategies (Monitoring and Evaluation)	Having the student check their solution steps (verify them); identifying strong and weak steps in the process.
	b. Causal Attribution	*Where did you succeed or struggle the most in today’s mathematics tasks, and why?*	Metacognitive Evaluation	Attributing success or failure not to chance, but to the strategy used, focus, and effort demonstrated (internal attribution)
Self-Reaction	Self-Satisfaction	*How satisfied are you with the effort and perseverance you demonstrated while tackling these math problems?*	Self-Reaction/Affective Monitoring	Assessing mathematics satisfaction and motivation in light of the effort and perseverance demonstrated.

## Appendix B. MLP-SRL Performance Phase Activities and Implementation Details

**Week/Topic**	**Activity**	**Strategy Used**	**Material**	**Mathematics Application**
**UNIT 1: SOLID GEOMETRY**				
1. Right Circular Cylinder	Graphical and Geometric Representation Analysis; Formula Deconstruction & Active Questioning; Step-by-Step Mathematics Modeling & Execution	Cognitive Strategies (Rehearsal, Organizational Strategies); Metacognitive Strategies (Planning, Monitoring, Evaluation)	Cardboard cylinder models, smart board, worksheets	The basic elements and net of a cylinder were examined using cardboard models and visuals displayed on the interactive whiteboard. The relationships between lateral surface area, total surface area, and volume were broken down into their components using guiding questions. Students identified the “given–requested” information in the problems, selected the appropriate relationship, and outlined the steps to solve them.
2. Right Circular Cone	Graphical and Geometric Representation Analysis; Formula Deconstruction & Active Questioning	Cognitive Strategies (Rehearsal, Elaboration, Organization)	Cardboard cone models, smartboard, worksheets	The basic elements and net of a cone were examined using cardboard models and visual representations on the smartboard. Students identified the radius, height, and slant height and explained the geometric relationships among these elements. The lateral and total surface area formulas were then broken down into their components, with guiding questions used to identify the geometric elements represented by the symbols. Students applied the formulas to examples while relating them to the cone’s geometric structure.
3. Sphere	Formula Deconstruction & Active Questioning; Graphical and Geometric Representation Analysis; Mathematics Error Analysis Challenge	Cognitive Strategies (Rehearsal, Elaboration, Organization); Critical Thinking	Sphere model, smartboard, worksheets	The surface area and volume formulas of a sphere were examined by breaking them into components. Using a sphere model and visual representations on the interactive whiteboard, students explored the relationships among radius, diameter, surface area, and volume and explained the formula symbols in relation to the geometric elements. The geometric relationships among the volumes of a sphere, cylinder, and cone were also examined using visual aids. Finally, students analyzed incorrect solutions, identified erroneous steps, explained the reasons for the errors, and constructed correct solutions.
4. Solid Geometry—Mixed Problems	Real-World Math Modeling (Contextual Problems); Collaborative Peer Problem-Solving Puzzles	Critical Thinking; Resource Management (Peer Learning)	Real-life problem cards, worksheets, and smartboard	Math modeling was conducted using real-life problems involving cylinders, cones, and spheres. Students were guided to identify the given and required information in the problems and select the appropriate geometric model and solution method. Complex problem-solving tasks were solved in small groups, and the solution methods were discussed and compared with peers.
**UNIT 2: PROBABILITY**				
5. Conditional Probability	Formula Deconstruction & Active Questioning; Step-by-Step Mathematics Modeling & Execution; Mathematics Error Analysis Challenge	Cognitive Strategies (Elaboration); Critical Thinking; Metacognitive Strategies (Planning, Monitoring, Evaluation)	Colored balls and bags, event cards, smartboard, worksheets	The conditional probability formula was broken down into its components. Using concrete examples, such as selecting colored balls from a bag, students examined how the sample space changes when an event occurs, guided by questions. They analyzed incorrect solutions to identify errors in the use of the sample space and conditional probability formula, then systematically constructed solution steps by identifying the given and required information.
6. Dependent and Independent Events	Graphical and Geometric Representation Analysis; Mathematics Error Analysis Challenge	Cognitive Strategies (Elaboration & Organization); Critical Thinking	Tree diagrams, incorrect solution cards, worksheets	Dependent and independent events were compared using tree diagrams and visual diagrams. By examining examples of incorrect solutions, the steps in which inappropriate operations or relationships were used were identified. Students were asked to explain the cause of the error and derive the correct solution.
7. Compound Events	Collaborative Peer Problem-Solving Puzzles; Step-by-Step Mathematics Modeling & Execution	Resource Management (Peer Learning); Cognitive Strategies (Rehearsal, Elaboration, Organizational Strategies)	Problem cards, worksheets, smartboard	Compound event problems, in which multiple events are considered together, were solved in small groups. During the problem-solving process, students were guided to analyze the problem, identify the components of the events, and establish the necessary probability relationships to develop the steps toward a solution. Group members discussed and compared different solution approaches and reached a consensus on the appropriate solution.
8. Experimental and Theoretical Probability	Graphical and Geometric Representation Analysis; Step-by-Step Mathematics Modeling & Execution	Cognitive Strategies (Rehearsal, Elaboration, Organizational Strategies); Metacognitive Strategies (Monitoring and Evaluation)	Dice, coins, data recording sheets, smartboard	Experiments involving dice rolls and coin tosses were conducted in the classroom; the results were recorded in data sheets and presented in tables and graphs. The experimental probability values were calculated and compared with the theoretical probability values. By examining how the results approached the theoretical probability as the number of experiments increased, the students’ mathematics interpreted the similarities and differences.
